# Experience of Using Transpedicular Osteosynthesis in Traumatic Spondylolisthesis of the Axis

**DOI:** 10.17691/stm2021.13.5.06

**Published:** 2021-10-29

**Authors:** I.V. Basankin, А.А. Giulzatyan, P.B. Nesterenko, А.B. Bagaudinov, D.А. Tayurski, М.L. Mukhanov

**Affiliations:** Head of Neurosurgical Unit No.3 Scientific Research Institute — Ochapovsky Regional Clinical Hospital No.1, Ministry of Health of Krasnodar Region, 167, 1^st^ Maya St., Krasnodar, 350086, Russia; Neurosurgeon Scientific Research Institute — Ochapovsky Regional Clinical Hospital No.1, Ministry of Health of Krasnodar Region, 167, 1^st^ Maya St., Krasnodar, 350086, Russia; Orthopedist Scientific Research Institute — Ochapovsky Regional Clinical Hospital No.1, Ministry of Health of Krasnodar Region, 167, 1^st^ Maya St., Krasnodar, 350086, Russia; Neurosurgeon Scientific Research Institute — Ochapovsky Regional Clinical Hospital No.1, Ministry of Health of Krasnodar Region, 167, 1^st^ Maya St., Krasnodar, 350086, Russia; Student, Medical Faculty Kuban State Medical University, 4 M. Sedina St., Krasnodar, 350063, Russia; Assistant, Department of Orthopedics, Traumatology and Military Field Surgery Kuban State Medical University, 4 M. Sedina St., Krasnodar, 350063, Russia

**Keywords:** spinal cord injury, upper cervical spine injury, Hangman’s fracture, transpedicular fixation, C_2_ fracture, Judet operation

## Abstract

**The aim of the study** was to assess the efficacy and safety of direct posterior transpedicular osteosynthesis in traumatic spondylolisthesis of C_2_ vertebra.

**Materials and Methods:**

The present study is an observational retrospective analysis of the results of surgical treatment of 19 patients operated on in 2014–2020 using the posterior transpedicular osteosynthesis technique with Herbert’s compression screws for a Hangman’s fracture type II according to Levine–Edwards classification. After the operation, the follow-up period lasted for 22 [10; 36] months.

**Results:**

The study group of patients (n=19) made 2.48% of all patients operated on for traumatic injury of the cervical spine (n=766) in the period from 2014 to 2020. In all cases, the surgical treatment was successful; there were no intraoperative complications in the form of damage to the vascular and nerve structures. The average duration of surgery was 70.8±24.5 min, and intraoperative blood loss was 92.9±41.8 ml. The length of hospitalization stay was 7 [5; 17] days. On the postoperative CT scans, no significant screw malposition (>2 mm) was found.

**Conclusion:**

Transpedicular osteosynthesis with compression screws in C_2_ traumatic spondylolisthesis is a safe and sparing operation with a short duration and insignificant blood loss. Thorough preoperative planning and knowledge of the anatomic landmarks make it possible to perform this operation effectively under the C-arm X-ray system control without any navigation system.

## Introduction

Fractures of the second cervical vertebra C_2_ (axis) often occur both in elderly and young age [1]. According to the data of the Sweden National Patient Register [2], the incidence of C_2_ fractures has doubled (from 3 to 6 per 100,000 population) from 1997 to 2014. According to literary sources [3–6], the incidence of such fractures makes 9–18% of all types of traumatic injuries of the cervical spine, the odontoid process (dens) fracture accounting for 35–78%, while C_2_ traumatic spondylolisthesis — for 11–25%.

Traumatic spondylolisthesis of the C_2_ vertebra, or the so-called Hangman’s fracture, was described for the first time by Schneider et al. in 1965 [7]. This type of injury occurs as a result of falls, diving, or road accident due to hyperextension and axial load acting on the axis [8].

This injury is treated with conservative and surgical methods aiming at achieving external or using a Philadelphia collar for 10‒14 weeks [9]. This technique is used only in neurologically intact and stable Levine– Edwards type I fractures, and in this case, good clinical and functional results with a high rate of fracture consolidation (up to 100%) have been noted [8].

Treating C_2_ traumatic spondylolisthesis by halo traction and reposition using Gardner‒Wells brackets is a well-known technique employed by many specialists. Vaccaro et al. [10] consider traction, reposition, and early rigid immobilization to be an effective method of treatment of type II and IIa axis traumatic spondylolisthesis in the majority of cases. At angulation >12°, a long immobilization period is required to achieve adequate consolidation [10]. However, this technique limits essentially the functionality of the extremely biomechanically important upper cervical spine. Tolerance to halo immobilization is especially reduced in elderly and aged people. In this regard, great attention in choosing a treatment technique is paid not only to reliable spondylosynthesis but to the functionality of the damaged segment as well.

Surgical management of the Hangman’s fracture includes several anterior and posterior stabilizing techniques. Depending on the fracture type, anterior cervical spondylodesis of C_2_‒C_3_, transpedicular osteosynthesis of C_2_, direct spondylosynthesis of C_2_‒C_3_ or С_1_–С_2_–С_3_ are performed [11–14]. All surgical methods except direct spondylosynthesis of the C_2_ vertebra block the spine motion segment and significantly restrict the range of motion in the upper cervical spine.

Direct transpedicular osteosynthesis of C_2_ in Hangman’s fracture was first mentioned in the work of Leconte [15] and further popularized by Judet [16]. This method was recognized to be a physiological operation as fixed is only the fracture, not the motion segment.

**The aim of the study** was to assess the efficacy and safety of the direct transpedicular osteosynthesis in traumatic spondylolisthesis of the C_2_ vertebra.

## Materials and Methods

The present study is an observational retrospective analysis of a series of cases (<50). Patients with isolated or combined traumatic spondylolisthesis of the C_2_ vertebra were the object of the investigation. The subject of the study was the technique of direct transpedicular osteosynthesis of the C_2_ vertebra with Herbert’s compression screws, its efficacy, and its influence on patients’ quality of life.

The results of surgical treatment of 19 patients operated on for Hangman’s fracture in the period from 2014–2020 using direct transpedicular osteosynthesis have been retrospectively analyzed. The study was performed following the requirements of Declaration of Helsinki (2013) and approved by the Ethical Committee of Scientific Research Institute — Ochapovsky Regional Clinical Hospital No.1 (Krasnodar, Russia). Written informed consent for participation in the study was obtained from each patient.

Inclusion criteria were as follows:

patient age 18‒70 years;

Levine–Edwards type II Hangman’s fracture.

Exclusion criteria implied:

abnormal development of C_2_ pedicles;

multifragmental fracture of the axis pedicles;

severe somatic comorbidity.

The distribution of the changes found in the spine was performed according to Levine–Edwards classification (to define the fracture type) [17] which was developed on the basis of classification proposed by Effendi [18] and is now most popular. It distinguishes four types of traumatic spondylolisthesis of the C_2_ vertebra:

type I — stable fracture, C_2_ body listhesis <3 mm, no angulation, C_2_‒C_3_ disc is intact;

type II — unstable injury, C_2_ traumatic antelisthesis >3 mm, angulation >11º, disruption of the posterior longitudinal ligament, vertical fracture line;

type IIa — unstable injury, no traumatic antelisthesis, fracture line is horizontal, not vertical, marked angulation >11º without antelisthesis;

type III — unstable injury, type I accompanied by bilateral facet joint dislocation.

To assess the patient’s condition objectively, neurological and clinical examination has been conducted; the degree of the spine injury was established using AIS (ASIA Impairment Scale), the intensity of the pain syndrome and quality of life was evaluated according to the visual analogue scale (VAS), and daily life activity restrictions due to neck-related pain were evaluated by the Neck Disability Index (NDI). Life activity restrictions were assessed in percentage terms as all patients omitted 1 or 2 items of the questionnaire (driving, reading). While interpreting the results, the following criteria were taken into consideration: 0–9% — no daily life restrictions; 10–29% — slight restriction; 30–49% — moderate; 50–69% — strong; >70% — full restriction. CT was used to visualize the pathologic substrate.

Direct transpedicular osteosynthesis of the C_2_ vertebra with Herbert’s compression screws was performed under C-arm X-ray system control without intraoperative navigation. The axial, frontal, and sagittal CT scans of the upper cervical spine were carefully analyzed in the preoperative period. To define a safe zone for screw insertion, the C_2_ diameter and height of the pedicles, their mediolateral and rostrocaudal angles, as well as variations of the spine artery course and the spinal canal diameter were taken into account.

Operative intervention was conducted in prone position of the patient with a soft roller under the chest, the head was fixed in the Mayfield clamp. A median incision was made in the projection of the C_0_‒C_3_ bodies using the massive spinous process of the C_2_ vertebra as a landmark. The nuchal ligament was exposed along with the spinous processes. Areas necessary for the insertion of the transpedicular screw were exposed subperiosteally, the inner edges of the C_2_ pedicles were also freed from the periosteum. The cranial edge of the axis arch served as a landmark for screw insertion and the superomedial pedicle border was accessible for visual control (the screw entrance angle was 15–20°, angulation — 10–15°). After the installation of the active drainages, the cervical muscles were sutured to the spinous process of the C_2_ vertebra. Sutures were put in the subcutaneous tissue and the skin.

The follow-up period after the operation was 22 months [10; 36].

**Statistical processing of the results**. The clinical results obtained were processed using the software package IBM SPSS 16.0. Numeric data are presented as median (Ме) and interquartile interval [25; 75] or arithmetic mean ± standard deviation (М±STD).

Since the number of patients in the general population was less than 50, the distribution of numeric values in relation to a sample differed essentially from the normal law of distribution (the hypothesis of normal distribution of variables in the population was checked using the Shapiro–Wilk test for normality; W=0.637; р<0.0001), nonparametric methods of statistical analysis and Wilcoxon signed-rank test were applied, and the level of statistical significance p<0.05 was accepted as a lower border of confidence.

## Results

The studied cohort of patients (n=19, 14 males and 5 females) makes 2.48% of the total number of the operated patients in the period from 2014 to 2020 for traumatic injury of the cervical spine (n=766). All patients were hospitalized to the neurological unit. Patients’ age was 22‒67 years. The traumas were caused by a traffic accident in 12 patients (63.2%) and fall from height in 7 patients (36.8%).

The main pre- and postoperative characteristics of the studied patients are presented in Table 1.

**Table 1:** Characteristics of patients

No.	Gender/ age	Trauma mechanism	Traumatic injury of other organs and systems	Neurological status according to AIS		Pains in cervical spine according to VAS			NDI	
				Before the operation	After the operation	Before the operation	3 days after the operation	3 months after the operation	Before the operation	3 мonths after the operation
1	М/23	Traffic accident	Mild brain injury, fracture of right radius	D	D	6	2	1	42	22
2	М/56	Traffic accident	Fractured ribs, lung contusion, hemothorax, fracture of left tibia diaphysis	E	E	6	1	1	35	4
3	М/62	Fall	—	E	E	7	2	1	48	7
4	F/22	Traffic accident	Brain injury of moderate severity	C	D	5	1	1	53	24
5	F/67	Traffic accident	—	D	E	7	2	0	54	2
6	F/34	Traffic accident	Fractured ribs, lung contusion, fracture of right humerus diaphysis	D	E	5	1	0	20	2
7	М/54	Traffic accident	Diacondylar fracture of right humerus	E	E	4	1	1	7	0
8	М/42	Traffic accident	Fractured ribs, pneumothorax, fracture of left fibula	E	E	8	3	1	65	6
9	F/48	Fall	Mild brain injury	E	E	6	2	1	39	4
10	М/44	Fall	Bilateral fracture of radii with displacement	E	E	6	2	1	35	0
11	М/51	Traffic accident	Isolated fracture of left lateral femoral condyle	E	E	7	3	1	53	6
12	М/28	Traffic accident	—	D	E	8	1	1	72	4
13	М/30	Traffic accident	Fractured ribs, hemothorax	D	D	8	0	3	62	15
14	F/45	Fall	Mild brain injury	D	E	8	0	0	55	7
15	М/65	Traffic accident	Fracture of left humerus and left femoral diaphysis	E	E	5	3	1	30	2
16	М/67	Traffic accident	—	E	E	7	2	0	55	0
17	М/45	Fall	Brain injury of moderate severity, compression fracture of Th_11_-vertebra type A1 according to AOSpine	С	E	6	2	1	47	4
18	М/57	Traffic accident	Fractured ribs, fracture of humeral surgical neck, right and left	E	E	5	3	1	25	6
19	М/22	Traffic accident	Fractured ribs	E	E	5	1	1	30	0

There were patients with combined pathology: 5 had brain injury of mild and moderate severity, 6 — fractured ribs and/or traumatic lung injury, 9 — fractures of the limbs, 1 had fracture of the Th_11_ vertebra.

Neurological symptoms have not been observed in 11 patients (57.9%) before and after the operative intervention. Complete regression of neurological symptoms by the AIS scale was noted in 5 people (in 4 from D to E, in 1 from C to E), in 1 patient there was incomplete regression of symptoms according to AIS (from C to D), no significant positive dynamics within the follow-up period was established in 2 patients (D by AIS).

**Figure 1.**
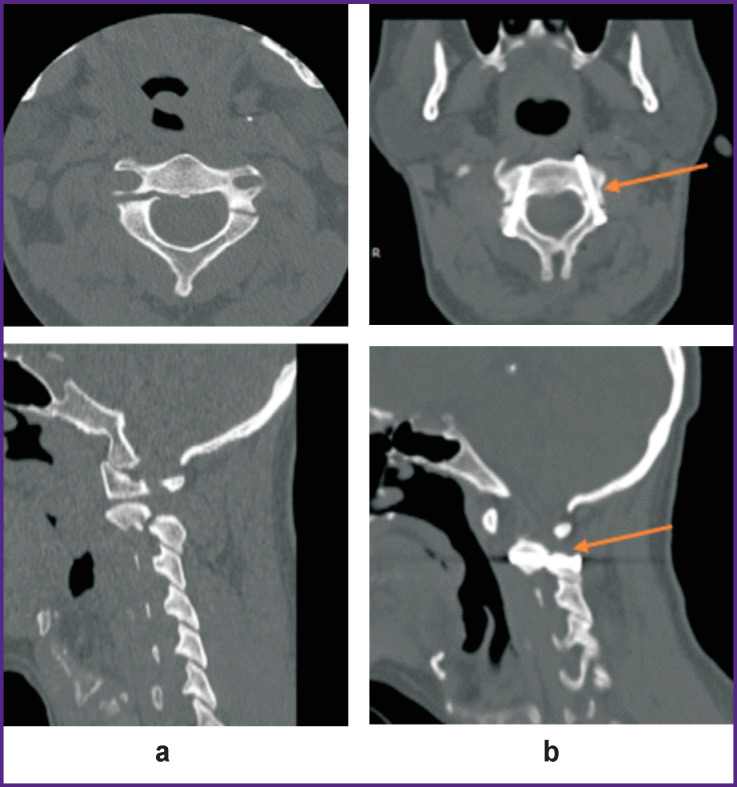
CT scans of the patient with traumatic spondylolisthesis of the C_2_ vertebra before (а) and after the operation (b) Arrows show compression screws

Surgical treatment was successful in all cases, intraoperative complications in the form of damage to the vascular and nerve structures have not been noted. Average duration of the operative intervention was 70.8±24.5 min, intraoperative blood loss was, on average, 92.9±41.8 ml. Hospital stay lasted for 5‒17 days. The postoperative CT examinations did not reveal any significant (>2 mm) malposition of the screws (Figure 1).

Average postoperative values according to VAS (3‒4 days and 3 months after the operation) and NDI (3  months after the operation) were statistically significantly better than the preoperative indices (Table 2).

**Table 2:** VAS and NDI indices before and after surgical treatment

Time of examination	VAS (points)		NDI (%)	
	Ме [25; 75]	Wilcoxon test; p	Ме [25; 75]	Wilcoxon test; p
Before operation	6 [5; 7]	Z_1_=–3.865	47.0 [32.5; 54.5]	
After operation:		p<0.001		— Z_2_=–3.824
3–4 days	2 [1; 3]	Z_2_=–3.853	—	p<0.001
3 months	1 [1; 1]	p<0.001	4.0 [2.0; 6.6]	

Here: Z_1_ — comparing data before the operation and 3–4 days after it; Z_2_ — comparing data before the operation and 3 months after it.

**Figure 2.**
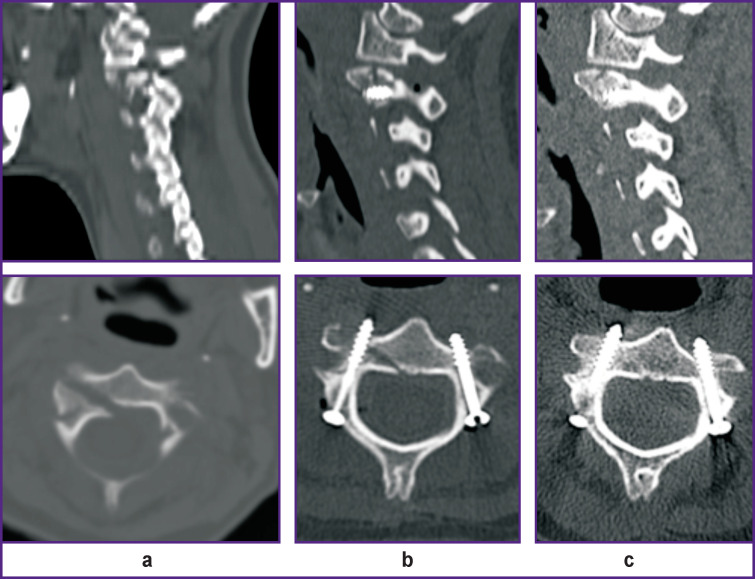
CT scans of the 22-year-old female patient with traumatic spondylolisthesis of the C_2_ vertebra: (а) preoperative sagittal and axial scans, fracture is visualized, diastasis between fragments on the right >3.5 mm; (b) 1 day after the operation, transpedicular screws are visualized, no marked malposition (>2 mm) is observed; (c) 6 months after the operation, fracture consolidation is noted

Further scheduled examinations did not show any negative dynamics in the patients’ state and functional restrictions, maximal follow-up period covered 36 months. The signs of complete fracture consolidation in the fixation area were noted no later than 24 months (Figure 2).

## Discussion

Despite the fact that traumatic spondylolisthesis of the C_2_ vertebra was first described in 1965, the choice of the optimal treatment tactics for this injury has remained debatable until now. According to the literary sources [19, 20], presently, surgical treatment is recommended in type II, IIa, III Levine–Edwards fractures. Adequate C_2_ spondylodesis restores the natural axis of the spine by anatomic listhesis reduction and thereby gives patients the possibility to lead an active life.

Several operative techniques of managing C_2_ traumatic listhesis are described in the literature [21], and C_2_‒C_3_ anterior cervical spondylodesis with the use of interbody implants and titanic plates has been historically considered to be traditional. However, it should be noted that this operation is rather difficult to perform technically because of a high risk of damage to the sublingual, facial, and superior laryngeal nerves and vascular structures as well. There is also a high risk of consolidation absence and pseudoarthrosis appearance after the operation since the intervention does not make it possible to operate on the injured C_2_ structures directly [22]. Due to the above reasons, this operation is not performed routinely for unstable Hangman’s fracture treatment.

It should be noted that both anterior cervical spondylodesis and spondylosynthesis of C_2_‒C_3_ and C_1_‒C_2_‒C_3_ restrict the mobility of the vertebral motor segment. In direct transpedicular osteosynthesis of the C_2_ vertebra, the amplitude of axis movements is preserved therefore this operation has been recognized physiological by many authors [8, 23]. Borne et al. [24] describe 18 patients with Hangman’s fracture. Of them, 13 underwent transpedicular osteosynthesis with compression screws. The authors have noted that this method is technically simple, effective, and safe giving good anatomic and functional results. Verheggen and Jansen [25] also report good results of surgical treatment of 13 patients with Levine– Edwards types II and IIa fractures who underwent transpedicular fixation. Boullosa with colleagues [26] have demonstrated the results of treating 10 patients with Hangman’s fracture types I and II according to Effendi classification; 9 patients were noted to have a good clinical result: fracture consolidation and complete regression of preoperative symptoms. ElMiligui et al. [23] performed surgical treatment using Judet approach to 15 patients with Levine–Edwards type II traumatic spondylolisthesis of the C_2_ vertebra and recognized the technique to be safe and effective. Hakało and Wroński [27] made a comparative analysis of the results of treating patients (n=17) with type II Hangman’s fracture (Effendi classification). Nine patients underwent anterior transoral spondylodesis of C_2_‒C_3_, eight patients were performed C_2_ transpedicular spondylosynthesis. The authors came to the conclusion that Judet’s technique is a safer, economically affordable, and effective method of treatment.

Liu et al. [28] published the work in which they have analyzed the results of treatment of 25 patients with Levine–Edwards types II, IIa traumatic C_3_ spondylolisthesis. Transpedicular fixation was done with modified screws. This modification is a Herbert’s-based double-threaded screw which can compress the fracture twice and from opposite sides. The authors obtained good results in all patients during 36±12-month-follow-up.

In our work, the operation according to Judet’s approach was performed in 19 patients with Levine–Edwards type II Hangman’s fracture, and good clinical and functional results were also obtained: postoperative VAS and NDI indices differed statistically significantly from those before the operation. We did not use this operation in types IIa, III fractures as we think that in case of the marked angulation there is an injury to C_2_‒C_3_ intervertebral disc and posterior longitudinal ligament, and therefore these fractures are unstable. In this case, C_2_‒C_3_ fixation will be more justified.

Undoubtedly, the Judet operation is a technically difficult task due to large individual variations of C_2_ pedicle size and the course of the spinal artery. A screw runs through the vertebral pedicle which is medially limited by the spinal canal and spinal cord and laterally by the spinal artery. The screw diameter is 3.5 mm, while the width of the pedicle through which it must pass is, on average, 5‒7 mm, at the same time, the screw passage is further complicated by the fracture with fragment disposition which changes the 3D anatomy of the posterior axis ring. According to the literature, the incidence of damage to the vital structures in transpedicular screw installation in the cervical spine varies from 11 to 66% [29]. Therefore, to install a screw successfully, careful preoperative preparation and three-dimensional presentation of vertebral pedicle morphology are necessary [30]. In our work, no significant malposition of the screws (>2 mm) after the operative treatment has been noted.

## Conclusion

Transpedicular osteosynthesis with compression screws is a sparing and safe operation with a short duration (70.8±24.5 min) and low level of blood loss (92.9±41.8 ml). The operation is difficult and must be performed with great caution to prevent possible severe complications. Thorough preoperative planning and knowledge of the anatomic landmarks will allow the effective performance of the operation under the C-arm X-ray control without navigation systems.
